# *Candida auris* MIC testing by EUCAST and clinical and laboratory standards institute broth microdilution, and gradient diffusion strips; to be or not to be amphotericin B resistant?

**DOI:** 10.1016/j.cmi.2024.10.010

**Published:** 2024-10-18

**Authors:** Maiken Cavling Arendrup, Shawn R. Lockhart, Nathan Wiederhold

**Affiliations:** 1 Unit for Mycology, Statens Serum Institut, Copenhagen, Denmark; 2 Department Clin Microbiol, Rigshospitalet, Copenhagen University, Copenhagen, Denmark; 3 Mycotic Diseases Branch, Centers for Disease Control and Prevention, CDC, Atlanta, GA, USA; 4 Department Pathology and Laboratory Medicine, University of Texas Health San Antonio, San Antonio, TX, USA

**Keywords:** Amphotericin, C. auris, Candidiasis, Gradient strip, MIC, Microbroth dilution, Reference

## Abstract

**Objectives:**

Reported amphotericin B resistance rates for *Candida auris* vary considerably. This may reflect clinically relevant differences in susceptibility, technical issues with testing, or adoption of a clinical breakpoint that bisects the wild-type population. We compared reference methods and two gradient diffusion strips using a shared *C. auris* strain collection.

**Methods:**

Forty *C. auris* strains from nine U.S. states and ≥3 clades were included. Fourteen MIC data sets were generated using European Committee on Antimicrobial Susceptibility Testing (EUCAST) E.Def 7.4, Clinical and Laboratory Standards Institute (CLSI) M27Ed4, Etest, and MIC gradient test strip (MTS, Liofilchem) MICs. MICs ≤1 mg/L were classified as susceptible.

**Results:**

EUCAST and CLSI amphotericin B MIC testing were robust across the included method variables. The modal MIC was 1 mg/L, distributions unimodal and narrow with similar geometric mean (GM)-MICs (0.745–1.072); however, susceptibility classification varied (0–28% resistance). Gradient diffusion strip testing resulted in wider and bimodal distributions for 8/9 data sets. If adopting, per manufacturer’s protocol, double inoculation for the Etest method, the modal MIC increased to 2–4 mg/L and resistance rates to 45–63% versus 25–30% with the single inoculation. The EUCAST, CLSI, Etest, and MTS strip MICs correlated to the optical density of drug-free control EUCAST wells, suggesting that some isolates grew better than others and that this was associated with MIC.

**Discussion::**

The EUCAST and CLSI MIC results were in close agreement, whereas the strip test showed wider and bimodal distributions with reader to reader and centre to centre variation. Our study adds to the concern for commercial MIC testing of amphotericin B against *C. auris* and suggests the current breakpoint leads to random susceptibility classification.

## Introduction

*Candida auris* is an emerging organism that has caused nosocomial infections and outbreaks in hospitals and other health care facilities globally. It is almost ubiquitously fluconazole resistant and can acquire echinocandin resistance within a few weeks of echinocandin therapy [1,2]. Reported amphotericin B resistance rates for *C. auris* vary considerably. Reviewing 42 publications from 2009 to 2019, Chaabane et al. [3] reported amphotericin B resistance rates ranging from 0% to 100% [3]. Since then, Lockhart et al. [4] reported 35% resistance among 54 isolates from Pakistan, India, South Africa, and Venezuela during 2012–2015, Helleberg et al. reported 0% resistance among 122 isolates from India [5], Ben Abid et al. [6] reported 0% resistance among 122 isolates from India, they also reported 79% amphotericin B resistance among 76 isolates in Qatar 2020–2021, whereas amphotericin B resistance rates varied between 33%, 59%, and 0–22.6% for Colombian isolates across studies and methods used for antifungal susceptibility testing [7–9].

These differences may reflect either clinically relevant differences in amphotericin B susceptibility potentially driven by clonality among outbreak strains, technical issues with testing or adoption of a clinical breakpoint (BP) that bisects the wild-type population. Indeed, Siopi et al. [10] recently found that the use of Sensititre YeastOne led to systematically higher MICs than Clinical and Laboratory Standards Institute (CLSI), with modal (range) MICs of 2 (1–8) versus 1 (0.25–1) mg/L, respectively. This led to amphotericin B resistance rates of 0 and 89% using the susceptibility breakpoint of 1 mg/L. Similarly, Kathuria et al. [11] found notable differences in MICs across CLSI, Vitek 2, and Etest results for 90 *C. auris* isolates, resulting in amphotericin B resistance of 16%, 100%, and 1%, respectively, depending on the method used.

We compared MIC results with two reference methods (European Committee on Antimicrobial Susceptibility Testing (EUCAST) and CLSI) and two brands of gradient diffusion strips (Etest and MTS strips) using a shared *C. auris* strain collection that includes at least three clades and is derived from multiple states in the United States.

## Materials and methods

Forty *C. auris* strains from nine U.S. states were included. Clade information was not available for six isolates. Of the 34 isolates that were typed, 20 were Clade I, 13 were Clade III, and one was Clade IV. The isolates were chosen in a way that only a single isolate came from any perceived geographic clone. These isolates were a fair representation of what can be expected across country-wide surveillance. Amphotericin B susceptibility was investigated using two reference methods (EUCAST E.Def 7.4 [12,13] and CLSI M27 Ed4 [14]) and two gradient diffusion strip tests (Etest [bioMérieux] and MTS [Liofilchem]). EUCAST MICs were determined three times on three separate days. Flat bottom plates (Nunc MicroWell, Nunclon Delta-treated MicroWell plates, catalogue no. 167008; Thermo Fisher Scientific) were prepared using “RPMI 1640 with MOPS and glucose pH 7” (SSI Diagnostica, catalogue no. 60984, SSI Diagnostica) and the International Organization for Standardization (ISO) method for drug dilution (two sets of plates) and serial dilution (one set of plates) [15]. MICs were read spectrophotometrically (490 nm wavelength and 90% growth inhibition) after 1 day of incubation.

CLSI MICs were determined twice on separate days using two internal lots of RPMI-1640 medium prepared on separate days from one purchased lot of medium (Sigma Aldrich; Catalogue No. R6504; Lot No. 2482886) and round bottom plates (Corning Costar Cell Culture-Treated plates, catalogue no. 3799, Fisher Scientific). Plates were prepared using the ISO method for drug dilution, as also published in the CLSI M27 Ed4 document. MICs were read visually using the endpoint of complete inhibition of visual growth after 1 day of incubation.

Etest MICs were determined in two laboratories: (a) consecutively as part of the ongoing routine testing, and (b) batched in another laboratory. In routine testing (at the CDC) a single reader read each individual Etest, but there were three people in total that read the MICs. All three were trained to read Etest by the same person and there was no discernible difference in the average MIC values across the readers (data not shown). In both laboratories, the standard procedure for Etest MIC determination was to inoculate agar once using a swab dipped once in the inoculum suspension, incubate at 35–37 °C, and read the MIC at 1 day (unless growth was insufficient). A third testing was performed strictly following Etest manufacturer instructions with double inoculation of the agar with a swab dipped twice in the inoculum suspension and plates read after 24 hours and 48 hours by three independent mycology laboratory staff members skilled in antifungal susceptibility testing.

MTS (Liofilchem) strip MICs were determined in batch testing. Suspensions of each *C. auris* strain (0.5 McFarland corresponding to ~1 × 10^6^ CFU/mL) were prepared. Sterile swabs were dipped once into the suspensions and used to inoculate the RPMI plates. The plates were briefly allowed to dry and then incubated at 35 °C for 1 day before reading the MICs.

For all MIC test methods, *Candidia. krusei* ATCC 6258 and *Candidia. parapsilosis* ATCC 220900 were included for quality control (QC). A summary of the retrieved QC results is presented in Table S1. Unfortunately, QC data for the consecutive testing was not retrievable, but QC strains were included every time a clinical isolate was tested, and the result was only accepted if both QC strains were within the QC range.

### Data management

EUCAST has not set breakpoints for amphotericin B against *C. auris*. However, MICs ≤1 mg/L are regarded as susceptible and MICs >1 as resistant for the more common species (clinical breakpoints for fungi v 10.0 available at the EUCAST website (EUCAST: breakpoints for antifungals) and are also the pragmatic EUCAST breakpoints for rare yeast [16]. The U.S. CDC has tentatively recommended a breakpoint of ≥2 mg/L as the resistant breakpoint for amphotericin B but has not made a recommendation for a susceptible breakpoint. The geometric mean (GM)-MICs were determined for each susceptibility test data set and compared. Spearman rank test (two tailed and 95% CI) was used to examine the correlation between growth in the positive control wells of EUCAST MICs (obtained using plates prepared with ISO dilution method for amphotericin B) and the MIC obtained with the different MIC tests (GraphPad Prism 10.0.2).

### Ethics

This is an anonymised global strain collection used for *in vitro* testing. Consent was not needed as no personal information was included.

## Results

**Fig. 1.**
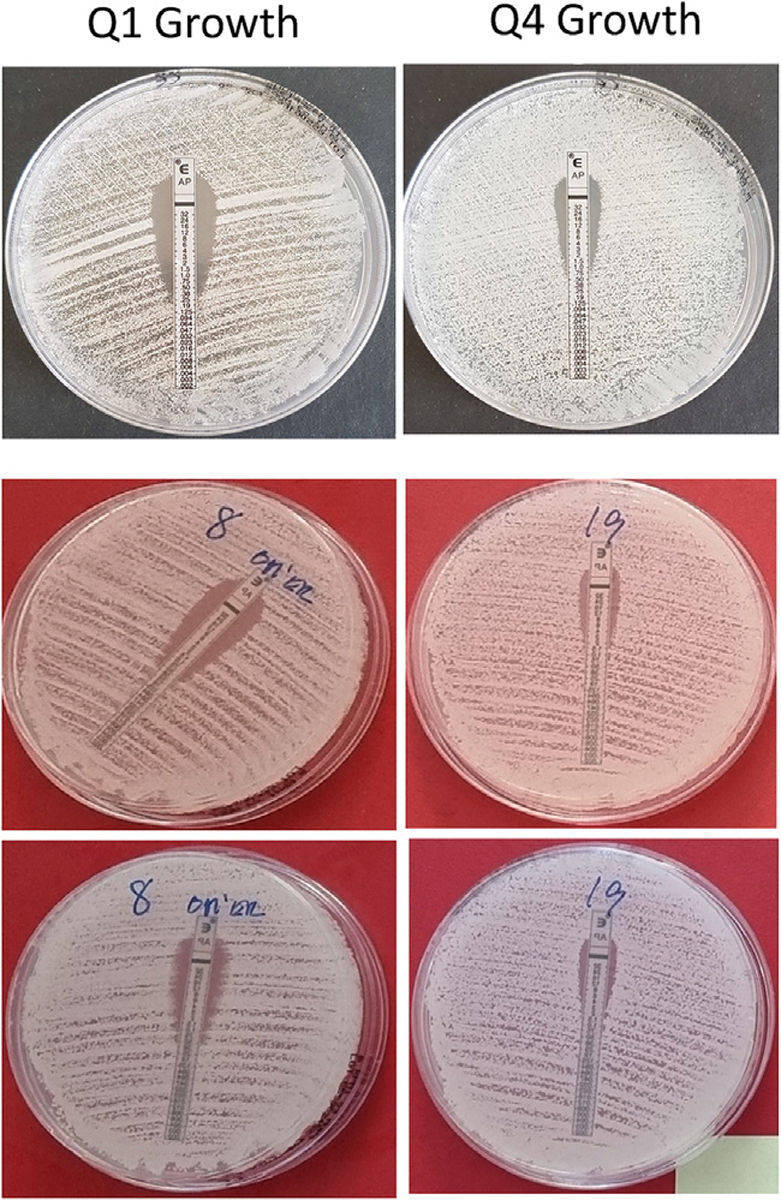
Etest examples for a Q1 growth (left) and Q4 growth (right) of *Candidia auris* strains using a single swabbing inoculation with 24 hours incubation (top panel), double swabbing inoculation with 24 hours (middle panel) and 48 hours incubation (lower panel).

EUCAST and CLSI amphotericin B MIC testing were robust across the included method variables (Table 1). The modal MIC was 1 mg/L for both methods, distributions unimodal and narrow, and GM-MICs similar (range 0.74–1.07). However, applying a BP of 1 mg/L led to differential susceptibility classification (0% for EUCAST and 8–28% resistance for CLSI). For EUCAST specifically, the MICs obtained using plates prepared with serial dilution (with pipette tip changes after columns 4 and 7) and ISO dilution for plate preparation were compared [15]. A significant correlation was found between the MICs for the three experiments (Fig. 1) and no pattern was observed between MIC and position of the isolate on the plate (inner or outer rows, Tables S2–S4).

Gradient diffusion strip testing with one swab inoculation resulted in slightly lower GM-MICs but wider MIC distributions and high resistance rates (25–30%) compared with the results for the reference methods. The MICs increased when the manufacturer’s recommendation to inoculate twice was followed, resulting in modal MIC of 2–4 mg/L and resistance rates of 45–58% and 60–63% when read after 24 hours and 48 hours, respectively. Seven of the eight Etest datasets were bimodal, with the first peak around 0.125–0.5 mg/L and the second larger peak around 1–4 mg/L, the exception being the double inoculation plates read by reader one after 24 hours incubation.

A significant and either moderate (Spearman r: 0.5– < 0.7) or strong (≥0.7) correlation was found for all combinations of the 14 MIC data set obtained with EUCAST, CLSI, and gradient diffusion strip testing, except for the CLSI BMD2 dataset, for which 36/40 MICs fell at 1 mg/L. (Fig. 1). Moreover, MICs from the EUCAST, CLSI, and gradient diffusion strips all correlated to the optical density of drug-free control in EUCAST wells, suggesting that some isolates grew better than others and that this was associated with MIC (Table 2 and Fig. 1).

## Discussion

The EUCAST and CLSI broth microdilution MIC results were in close agreement with each other across the potential sources of variation included in the study. Thus, CLSI MIC testing was robust across two different batches of broth medium; EUCAST MIC testing was not influenced by choice of plate preparation method (using the ISO scheme for drug dilution, or serial dilution in the plate with two tip changes) nor by the position on the plate, in contrast to what can be observed for isolates from other species that grow less well in the middle rows of the plate [15]. The gradient diffusion strips generated wider and bimodal distributions with reader to reader and centre to centre variation. This was particularly notable for the consecutive Etest MIC testing data set, both compared with the batched Etest and MTS strip testing and to the reference methods. Greater variation is expected during consecutive testing; however, in this case MICs covered nine dilutions compared with four for batched testing, which may compromise reproducibility and correct susceptibility classification. Of note, all QC MICs fell within the target (EUCAST) and within the range (remaining tests), meaning that variation was not captured by QC strains (Table S1). Therefore, our results add to the concern on amphotericin B susceptibility testing using commercial methods [10,11].

A correlation was found between MICs for the isolates by the different methods. This may suggest a true differential susceptibility, or it may relate to differential growth rates of the strains. MICs in general depend on growth, the classical example being the higher MICs obtained for plates read after two rather than 1 day of incubation. This is particularly true for diffusion tests where the endpoint is not determined as a fixed proportion of the growth for the growth control for that particular isolate and incubation time. Of note, the mean optical density of positive control wells in EUCAST plates correlated to the MIC not only for EUCAST results but also for CLSI, Etest, and MTS results, suggesting that some isolates grew better than others in all three labs and that this was associated with the MIC. This raises the question of whether this MIC variation, that in reference testing is discrete, correlated to clinical efficacy or is simply a reflection of growth *in vitro*.

Few studies have investigated underlying mechanisms in *C. auris* isolates classified as amphotericin B resistant. The biosynthesis of ergosterol in *Candida* cells involves a number of enzymatic steps encoded by *erg6, erg11, erg24, erg25, erg26, erg27, erg2, erg3, erg5,* and *erg4* in sequential order [17]. Amphotericin B resistance in *Candida* spp. has been associated with mutations in *erg2* [18,19] and *erg6* [20] and with combined mutations in *erg11* and in *erg3* or *erg5* [18–22]. In *C. auris*, Carolus et al. [23] found that simultaneous emergence of nonsense mutations in *erg3* and *erg11* in experimentally evolved multidrug resistant *C. auris* led to decreased amphotericin B susceptibility (MIC 2 mg/L) and that a mutation in *mec3*, a gene involved in DNA damage homeostasis, further increased polyene MIC (4 mg/L) as determined by the CLSI method. Ben Abid et al. [6] reported a unique premature stop codon in *erg3* in an isolate amphotericin B resistant (MIC 2 mg/L) with Vitek2 testing; however, nine isolates with higher amphotericin B MICs (4–≥16 mg/L) did not harbour this or other known amphotericin B associated mutations [6]. Escandón et al. [24] reported a significant association between four nonsynonymous mutations and elevated amphotericin B Etest MIC. One of these was located in a gene predicted to encode for a transcription factor similar to FLO8, which in *C. albicans* has been associated with biofilm formation and amphotericin B resistance [25], whereas another mutation was located in a gene predicted to encode for a membrane transporter of unknown importance for amphotericin B susceptibility. Jacobs et al. whole genome sequenced 19 consecutive isolates, all of which were classified as amphotericin B resistant by colorimetric susceptibility testing using the Sensititre YeastOne assay and four of which were resistant by Etest (MIC 2 mg/L) [26]. But no known genetic causes for resistance were found.

One of the limitations of this study is that resistance to amphotericin B may be epigenetic. If this is the case, passage of the isolates in the laboratory with no drug pressure may cause the MIC to decrease. This could also account for some of the discrepancies between laboratories. We were also unable to determine whether any of the patients from whom these isolates were collected had been treated with amphotericin B.

Breakpoints are typically set statistically to encompass 97.5–99% of the modelled wild-type MIC distribution, resulting in a breakpoint that is 1–2 two-fold dilutions above the modal MIC. In all 14 datasets in this study, the modal MIC was too close to a susceptibility BP of ≤1 mg/L. This will inevitably lead to variable MIC classification, as found here, with resistance rates varying from 0% to 63% for the same strain collection despite the modal MIC being similar. Consequently, from a technical point of view, either *C. auris* should be regarded as susceptible (and the BP set at 2 mg/L), or the entire population should be deemed resistant to amphotericin B to avoid random classification. A BP of 2 mg/L (one dilution higher than the modal MIC for EUCAST and CLSI would for this dataset result in the following numbers of “resistant isolates”: Etest consecutive: 2/40 (5%), Etest batched: 0/40 (0%), and MTS batched: 2/40 (5%) provided single swab inoculation is adopted, and 0% for all five EUCAST and CLSI runs. Our proposal in the absence of solid data linking the majority of elevated MICs to molecular resistance mechanisms would be to regard isolates with MIC ≤2 mg/L as wild-type isolates for which amphotericin B is a valid option but not the first treatment choice unless the organism is echinocandin resistant.

## Supplementary Material

supplementary appendix

## Figures and Tables

**Table 1:** Overview of amphotericin B MICs against 40 *Candidia auris* isolates obtained by EUCAST and CLSI reference methods, and by Etest and MTS gradient strips

Test method	MIC (mg/L)										% “R”	GM-MIC	Total
	0.03	0.06	0.125	0.25	0.5	1	2	4	8	16			

Reference methods													
EUCAST ISO-1					15	25^a^					0%	0.77	40
EUCAST ISO-2				1	15	24^a^					0%	0.74	40
EUCAST serial					14	26^a^					0%	0.78	40
CLSI medium #1					7	22^a^	11				28%	1.07	40
CLSI medium #2					1	36^a^	3				8%	1.04	40
Etest													
One swab inoc, consecutive testing	1	5^b^	5^b^	3	7	9^a^	8	1		1	25%	0.51	40
One swab inoc, batched testing				11^b^	4	13^a^	12				30%	0.78	40
Two swab inoc 24 h person R1					3	6	17^a^	1			45%	0.82	40
Two swab inoc 24 h person R2				9^b^	6	4	15^a^	6			53%	1.37	40
Two swab inoc 24 h person R3			1	11^b^	3	2	14^a^	9			58%	1.05	40
Two swab inoc 48 h person R1				1	13^b^	2	15^a^	9			60%	1.74	40
Two swab inoc 48 h person R2					13^b^	2	6	18^a^	1		63%	1.07	40
Two swab inoc 48 h person R3					11^b^	4	5	18^a^	2		63%	1.87	40
MTS													
MTS		2	10^b^	0	1	10	14^b^	2			41%	0.69	39

CLSI, Clinical and Laboratory Standards Institute; Inoc, inoculation.

a Modal MICs.

b The additional peak in bimodal distributions.

**Table 2:** Method specific MICs (mg/L) dependent of the growth rate quartiles (Q1–4) determined as Optical Density (OD) in EUCAST drug-free positive control wells (prepared following the ISO dilution scheme) during testing in laboratory 2

	EUCAST ISO MIC^a^			CLSI BMD1 MIC^b^				MTS MIC^c^					
	0.5	1		0.5	1	2		0.06	0.125	0.5	1	2	4
		
Q1	9	1	Q1	4	6		Q1	2	6			1	
Q2	5	5	Q2	3	4	3	Q2		4	1	1	4	
Q3		10	Q3		6	4	Q3				5	3	2
Q4	1	9	Q4		6	4	Q4				4	6	



	Etest Routine MIC^d^										Etest Batched MIC^e^			
	0.03	0.06	0.125	0.25	0.5	1	2	4	16		0.25	0.5	1	2

Q1	1	3	4		1				1	Q1	7	2	1	
Q2		2	1	2	2	2	1			Q2	4	1	1	4
Q3					3	2	4	1		Q3			7	3
Q4				1	1	5	3			Q4		1	4	5

CLSI, Clinical and Laboratory Standards Institute; OD, optical density.

EUCAST ISO-1 OD ranges for the quartiles were as follows: Q1: 0.325–0.363, Q2: 0.363–0.426, Q3: 0.426–0.467, and Q4: 0.468–0.506.

a EUCAST ISO (laboratory 2) versus EUCAST ISO pos control OD quartile.

b CLSI BMD1 (Laboratory 3) versus EUCAST ISO pos control OD quartile.

c MTS strip test (laboratory 3) versus EUCAST ISO pos control OD quartile.

d Etest MIC (laboratory 1) performed consecutively in the routine versus EUCAST ISO Pos Control OD quartile.

e Etest MICs (laboratory 2) run batched and set as consensus MICs across three technicians.
