# Supplementary material for: Candida auris MIC testing by EUCAST and clinical and laboratory standards institute broth microdilution, and gradient diffusion strips; to be or not to be amphotericin B resistant?
Source: Clin Microbiol Infect. Author manuscript; Available in PMC 2025 Mar 24. (PMC11931498; doi:10.1016/j.cmi.2024.10.010)
Supplement: supplementary appendix [file NIHMS2063125-supplement-supplementary_appendix.docx]

**Supplementary Material**

**Supplementary Table 1.** Summary of MIC results for quality control strain.

|  | *C. krusei* ATCC 6258 | | *C. parapsilosis* ATCC 22019 | |
| --- | --- | --- | --- | --- |
|  | n. tests | MIC or GM-MIC(Range) | n. tests | MIC or GM-MIC(Range) |
| EUCAST ISO-1 SSI | 8 | 0.5 (0.5) | 8 | 0.30 (0.25-0.5) |
| EUCAST ISO-2 SSI | 8 | 0.46 (0.25-0.5) | 8 | 0.39 (0.25-0.5) |
| EUCAST Serial SSI | 8 | 0.46 (0.25-0.5) | 8 | 0.27 (0.25-0.5) |
| CLSI BMD#1 | 1 | 1 | 1 | 0.5 |
| CLSI BMD#2 | 1 | 1 | 1 | 0.5 |
| Etest USA rounded daily | NA | NA | NA | NA |
| Etest SSI rounded | 1 | 0.5 | 1 | 0.25 |
| Etest manufac 24h rounded | 1 | 0.25 | 1 | 0.125 |
| Etest manufac 24h rounded | 1 | 0.25 | 1 | 0.125 |
| Etest manufac 24h rounded | 1 | 0.25 | 1 | 0.125 |
| Etest manufac 48h rounded | 1 | 0.5 | 1 | 0.5 |
| Etest manufac 48h rounded | 1 | 0.5 | 1 | 0.25 |
| Etest manufac 48h rounded | 1 | 0.5 | 1 | 0.25 |
| MTS rounded | 2 | 1 (1) | 2 | 0.71 (0.5-1) |

QC targets and (ranges) ATCC 6258/ATCC 22019: EUCAST, 0.25-5 (0.125-1)/0.25-5 (0.125-1); CLSI, 0.5-2/0.25–2; Etest (0.5-2)/(0.25-1); MTS, 0.5-2/0.25–1.

**Supplementary Table 2.** MICs for the 40 *C. auris* isolates and three CBS reference strains CBS 12372, CBS 12373 and CBS 10913 on plates prepared by the ISO dilution method as a function of the MICs for the same isolates determined on plates prepared using the serial dilution method. A correlation between MICs obtained with the two plate preparation methods were observed.

| **MICs (mg/L) on plates prepared with ISO dilution** | **MICs (mg/L) on plates prepared with serial dilution** | | | | | | |
| --- | --- | --- | --- | --- | --- | --- | --- |
|  | 0.25 | | | 0.5 | | 1 | |
| 0.25 | | 1 |  | |  | |  |
| 0.5 | |  | 14 | | 2 | |  |
| 1 | |  | 1 | | 25 | |  |

**Supplementary Table 3.** EUCAST MICs as a function of the position on the plate (inner or outer rows) for 40 *C. auris* isolates and three CBS reference strains CBS 12372, CBS 12373 and CBS 10913 MIC tested in three separate experiments using plates prepared by the ISO dilution method (*n*=2) and serial dilution with pipette tip change after well 4 and 7. Heat mapping is used to highlight cells with more values. No correlation between position on the plate and MICs was observed.

| **Row on plate** |  | **ISO-1** |  |  |  |  | **ISO-1** |  |  |  |  | **Serial** |  |  |
| --- | --- | --- | --- | --- | --- | --- | --- | --- | --- | --- | --- | --- | --- | --- |
|  | **0.25** | **0.5** | **1** | **Total** |  | **0.25** | **0.5** | **1** | **Total** |  | **0.25** | **0.5** | **1** | **Total** |
| A |  | 2 | 4 | 6 |  | 1 | 3 | 2 | 6 |  |  | 3 | 3 | 6 |
| B |  | 3 | 3 | 6 |  |  | 3 | 3 | 6 |  |  |  | 6 | 6 |
| C | 1 | 3 | 2 | 6 |  |  | 2 | 4 | 6 |  |  | 2 | 4 | 6 |
| D |  | 2 | 3 | 5 |  |  | 2 | 3 | 5 |  |  | 2 | 3 | 5 |
| E |  | 1 | 4 | 5 |  | 1 | 1 | 3 | 5 |  | 1 | 2 | 2 | 5 |
| F |  | 2 | 3 | 5 |  |  | 1 | 4 | 5 |  |  | 2 | 3 | 5 |
| G |  | 2 | 3 | 5 |  |  | 2 | 3 | 5 |  |  | 2 | 3 | 5 |
| H |  | 1 | 4 | 5 |  |  | 2 | 3 | 5 |  |  | 2 | 3 | 5 |
| **Totals** | **1** | **16** | **26** | **43** |  | **2** | **16** | **25** | **43** |  | **1** | **15** | **27** | **43** |

**Supplementary Table 4.** MICs for the 40 *C. auris* isolates and three CBS reference strains CBS 12372, CBS 12373 and CBS 10913 on two sets of plates prepared by the iso dilution method dependent on the position on the plate. Heat mapping is used to highlight cells with more values. No correlation between position on the plate and MICs is observed.

| **ISO-1 by Row on the plate** | **MIC (mg/L)** | | | **Total** |  | **ISO-2 by Row on the plate** | **MIC (mg/L)** | | | **Total** |
| --- | --- | --- | --- | --- | --- | --- | --- | --- | --- | --- |
|  | 0.25 | 0.5 | 1 |  |  |  | 0.25 | 0.5 | 1 |  |
| A |  | 2 | 4 | 6 |  | A | 1 | 3 | 2 | 6 |
| B |  | 3 | 3 | 6 |  | B |  | 3 | 3 | 6 |
| C | 1 | 3 | 2 | 6 |  | C |  | 2 | 4 | 6 |
| D |  | 2 | 3 | 5 |  | D |  | 2 | 3 | 5 |
| E |  | 1 | 4 | 5 |  | E | 1 | 1 | 3 | 5 |
| F |  | 2 | 3 | 5 |  | F |  | 1 | 4 | 5 |
| G |  | 2 | 3 | 5 |  | G |  | 2 | 3 | 5 |
| H |  | 1 | 4 | 5 |  | H |  | 2 | 3 | 5 |
| **Total** | **1** | **16** | **26** | **43** |  | **Total** | **2** | **16** | **25** | **43** |
